# ASSISTED LIVING IMMUNIZATION PROJECT

**DOI:** 10.1093/geroni/igz038.1828

**Published:** 2019-11-08

**Authors:** Maureen S Beck

**Affiliations:** McGovern Medical School at UTHealth, Houston, Texas, United States

## Abstract

Influenza immunization rates in the United States hover around 60% for people 65 years of age and older. Healthy People 2020 set a target rate of 90% for flu immunization. The influenza vaccine can reduce disease and expense with approximately 7 million flu related diseases, 109,000 hospital admissions, and 8,000 deaths prevented. A local assisted living had an unusually low influenza immunization rate last year of 40%. The goal of the quality improvement project was to increase the rate of influenza immunizations in this facility. The rational was that by providing education, promoting the event, and increasing direct communication with families and residents through phone call and emails the rate would improve. A collaborative project was begun in the summer of 2018. Approval was received by IRB and the facility administrators. Plans were made for two presentations to family and residents, promotional advertisement, family communication, and a scheduled flu day with the pharmacy. The presentation provided flu history and facts and myths about the immunization. Correct answers were rewarded with bookmarks with flu information. The assisted living director provided follow-up immunization data. Compared to the previous year, the immunization rate increased to 90%, counting immunizations on flu shot day and records of outside immunizations. Data concerning flu incidence from the 2017-2018 and 2018-2019 seasons will be requested to determine project impact. A partnership between primary care providers and long term care facilities focused on improving standards or meeting metrics can enhance care for your patient and the community.

